# Central venous-to-arterial PCO_2_ difference as a marker to identify fluid responsiveness in septic shock

**DOI:** 10.1038/s41598-021-96806-6

**Published:** 2021-08-26

**Authors:** Boulos Nassar, Mohamed Badr, Nicolas Van Grunderbeeck, Johanna Temime, Florent Pepy, Gaelle Gasan, Laurent Tronchon, Didier Thevenin, Jihad Mallat

**Affiliations:** 1 University of Iowa Hospitals and Clinics, Pulmonary and Critical Care Division, Iowa City, USA; 2 Department of Critical Care Medicine, Critical Care Institute, Cleveland Clinic Abu Dhabi, Al Maryah Island, Abu Dhabi, United Arab Emirates; 3 Department of Anesthesiology and Critical Care Medicine, Centre Hospitalier du Dr. Schaffner, Lens, France; 4 Cleveland Clinic Lerner College of Medicine of Case Western Reserve University, Cleveland, OH USA; 5 Normandy University, UNICAEN, ED 497, Caen, France

**Keywords:** Medical research, Translational research

## Abstract

Defining the hemodynamic response to volume therapy is integral to managing critically ill patients with acute circulatory failure, especially in the absence of cardiac index (CI) measurement. This study aimed at investigating whether changes in central venous-to-arterial CO_2_ difference (Δ-ΔPCO_2_) and central venous oxygen saturation (ΔScvO_2_) induced by volume expansion (VE) are reliable parameters to define fluid responsiveness in sedated and mechanically ventilated septic patients. We prospectively studied 49 critically ill septic patients in whom VE was indicated because of circulatory failure and clinical indices. CI, ΔPCO_2_, ScvO_2_, and oxygen consumption (VO_2_) were measured before and after VE. Responders were defined as patients with a > 10% increase in CI (transpulmonary thermodilution) after VE. We calculated areas under the receiver operating characteristic curves (AUCs) for Δ-ΔPCO_2_, ΔScvO_2_, and changes in CI (ΔCI) after VE in the whole population and in the subgroup of patients with an increase in VO_2_ (ΔVO_2_) ≤ 10% after VE (oxygen-supply independency). Twenty-five patients were fluid responders. In the whole population, Δ-ΔPCO_2_ and ΔScvO_2_ were significantly correlated with ΔCI after VE (r =  − 0.30, *p* = 0.03 and r = 0.42, *p* = 0.003, respectively). The AUCs for Δ-ΔPCO_2_ and ΔScvO_2_ to define fluid responsiveness (increase in CI > 10% after VE) were 0.76 (*p* < 0.001) and 0.68 (*p* = 0.02), respectively. In patients with ΔVO_2_ ≤ 10% (n = 36) after VE, the correlation between ΔScvO_2_ and ΔCI was 0.62 (*p* < 0.001), and between Δ-ΔPCO_2_ and ΔCI was − 0.47 (*p* = 0.004). The AUCs for Δ-ΔPCO_2_ and ΔScvO_2_ were 0.83 (*p* < 0.001) and 0.73 (*p* = 0.006), respectively. In these patients, Δ-ΔPCO_2_ ≤ -37.5% after VE allowed the categorization between responders and non-responders with a positive predictive value of 100% and a negative predictive value of 60%. In sedated and mechanically ventilated septic patients with no signs of tissue hypoxia (oxygen-supply independency), Δ-ΔPCO_2_ is a reliable parameter to define fluid responsiveness.

## Introduction

Hemodynamic optimization through fluid resuscitation is commonly used in critically ill patients with tissue hypoperfusion. The goal of volume expansion (VE) is to raise cardiac output and oxygen supply to improve tissue oxygenation. Recognizing patients who would benefit from VE remains challenging^1^. Identifying such patients is often dependent on measuring cardiac output^2,3^. Echocardiography is a skill that has made strides but not fully penetrated all critical care areas; it is limited by ultrasound availability and poor echogenicity, especially in mechanically ventilated patients. Passive leg raising test and end-expiratory occlusion methods also necessitate cardiac output measurement to asses fluid responsiveness^4,5^. Other measurements such as pulse pressure and stroke volume variations require specific technologies^6^ or are restricted to certain patient populations^7^. With these limitations, defining fluid responsiveness without cardiac output measurement would be of great help to the clinician at the bedside.

Venous-to-arterial CO_2_ tension difference reflects the balance between CO_2_ production and CO_2_ delivery to the lungs, a surrogate of the cardiac output^8,9^. Opposing changes over time in central venous-to-arterial CO_2_ tension difference (ΔPCO_2_) and cardiac output were reported in septic shock patients^10–12^. In post-cardiac surgery sedated and mechanically ventilated patients, Yazigi et al. observed a significant inverse correlation between changes in ΔPCO_2_ (Δ-ΔPCO_2_) and changes in cardiac index (ΔCI) induced by VE^13^. Moreover, in the same population, changes in central venous oxygen saturation (ΔScvO2) after VE were a reliable parameter to define fluid responsiveness^14^. However, data in the septic population is lacking.

In situations with tissue hypoxia, the increase in cardiac output and oxygen delivery (DO_2_) after VE would result in an increase in CO_2_ production (VCO_2_) and oxygen consumption (VO_2_) (oxygen supply dependency). These metabolic changes might confound fluctuations in arteriovenous O_2_ and CO_2_ parameters attributed solely to circulatory changes. This might reduce the changes in ΔPCO_2_ and ScvO_2_ induced by VE. Clinical studies have shown that the ratio of ΔPCO_2_ over the arterial-to venous oxygen content (ΔPCO_2_/ΔContO_2_) was a good indicator of oxygen supply dependency (tissue hypoxia) in critically ill patients^15,16^. This indicator could perhaps be used to identify such patients and guide the usage of CO_2_ and oxygen gaps.

Therefore, our study aimed to investigate: (1) if Δ-ΔPCO_2_ and ΔScvO_2_ are reliable parameters to identify fluid responsiveness in overall sedated and mechanically ventilated septic patients; (2) if the reliability of these parameters would be better in the sub-group of patients with no tissue hypoxia, defined as the absence of an increase in VO_2_ induced by a rise in DO_2_ after VE (oxygen supply independency); (3) if baseline ΔPCO_2_ /ΔContO_2_ ratio is a good predictor of tissue hypoxia. Such measurements are readily available in these patients with central venous and arterial catheters.

## Materials and methods

This prospective and observational study was conducted in a single, mixed medical and surgical adult intensive care unit (ICU) between April and December 2017. The study was approved by the local institutional ethics committee (Comité d’Ethique du centre hospitalier du Dr. Shaffner de Lens). Informed consent was obtained from the next of kin of each patient. All experiments were performed in accordance with relevant guidelines and regulations.

### Patients

We studied mechanically ventilated patients with sepsis^17^ for whom the attending physician decided to give VE due to the presence of at least one clinical sign of tissue hypoperfusion^17^ as previously described^15^: (a) systolic arterial pressure < 90 mmHg, mean arterial pressure < 65 mmHg, or the requirement for vasopressor administration; (b) skin mottling; (c) lactate levels > 2 mmo/l; or urinary output < 0.5 ml/kg/h for ≥ 2 h. Also, patients had to have a PiCCO device (PiCCO, Pulsion Medical System, Munich, Germany) as part of routine management of persistent signs of inadequate tissue perfusion in our ICU. Exclusion criteria were: pregnancy, age < 18 years old, moribund, and risk of fluid loading-induced pulmonary edema.

### Measurements

Demographic data, acute circulatory failure etiology, the Simplified Acute Physiology Score (SAPS) II, and the Sequential Organ Failure Assessment (SOFA) scores were obtained on the day of enrollment. CI was obtained with the PiCCO device by central venous injections of 20 ml of iced 0.9% saline solution and recorded as the average of the three measurements.

Arterial and central venous blood gases were measured using the GEM Premier 4000 (Instrumentation Laboratory Co, Paris, France). The central venous blood was collected from a central venous catheter with the tip confirmed to be in the superior vena cava, near or at the right atrium, by radiograph as previously described^15^. ∆PCO_2_ was calculated as the difference between the central venous carbon dioxide tension and the arterial carbon dioxide tension. The arterial oxygen content was calculated as CaO_2_ (ml) = 1.34 × Hb (g/dl) × SaO_2_ + 0.003 × PaO_2_ (mmHg), where SaO_2_ is the oxygen saturation of arterial blood, Hb the hemoglobin concentration, and PaO_2_ the arterial oxygen tension. The central venous oxygen content was calculated as CcvO_2_ (ml) = 1.34 × Hb (g/dl) × ScvO_2_ + 0.003 × PcvO_2_ (mmHg), where PcvO_2_ is the central venous oxygen tension. ΔContO_2_ (ml) was calculated as CaO_2_ − CcvO_2_. DO_2_ (ml/min/m^2^) was calculated as CaO_2_ × CI × 10. VO_2_ (ml/m^2^) was calculated as CI × ΔContO_2_ × 10. Oxygen extraction was defined as OE = VO_2_/DO_2_. We also calculated the ∆PCO_2_/ΔContO_2_ ratio.

### Study protocol

A first set of hemodynamic and oxygen-CO_2_ derived variables measurements was performed at baseline, including heart rate (HR), systemic arterial pressure, CI (thermodilution), DO_2_, VO_2_, ScvO_2_, arterial lactate level, and ∆PCO_2_. A 500 ml of colloid solution (4% Human serum albumin, Vialebex®; LFB) was administered to the patient over 15 min via a specific venous line. The same set of measurements was repeated immediately after the end of VE infusion. Ventilation parameters and infusions of norepinephrine and sedation drugs were remained unchanged during the VE.

Changes in hemodynamic and oxygenation variables were expressed as relative changes (([parameter after volume expansion − parameter before volume expansion]/parameter before volume expansion) × 100).

### Statistical analysis

Patients in whom 500-ml VE increased thermodilution-derived CI > 10% were defined as responders and the remaining ones as non-responders. Also, patients were divided into two subgroups according to their increases in VO_2_ (≤ or > 10%) induced by VE. All data are expressed as mean ± SD, or as median [25–75%, interquartile range, (IQR)], as appropriate. The normality of data distribution was assessed using the Shapiro–Wilk test. Comparisons of values between responders and non-responders were performed by two-tailed Student’s t test, or Wilcoxon rank-sum test, as appropriate. Pairwise comparisons between different study times were assessed using paired Student’s t test or Wilcoxon signed-rank test, as appropriate. Analysis of categorical data was performed using the Chi2 and Fisher’s exact tests. Linear correlations were tested by using the Pearson or the Spearman test, as appropriate.

Receiver operating characteristic (ROC) curves were constructed to evaluate the ability of each parameter to predict fluid responsiveness after fluid challenge. The AUCs were compared using the nonparametric technique described by DeLong et al.^18^. Previously, we have shown that the upper 95% confidence interval values of the least significant changes (LSC), which are the minimum changes that needed to be measured by a laboratory analyzer in order to recognize a real change of measurement, for ΔPCO_2_ and ScvO_2_ were 36.5% and 5.0% respectively^19^. Sensitivity, specificity, positive predictive value (PPV), negative predictive value (NPV), positive likelihood ratio (LR^+^), negative likelihood ratio (LR^−^), and their 95% confidence intervals were calculated for Δ-ΔPCO_2_ and ΔScvO_2_.

Variables are usually considered of good clinical tool (having good discriminative property tests) when the inferior limits of the 95% confidence interval of their AUC are more than 0.75^20^. For this purpose, 43 patients would be sufficient for a power of 80% and an alpha risk of 0.05. Statistical analysis was performed using STATA 14.0 (StataCorp LP, College Station, Texas, USA). *p* < 0.05 was considered statistically significant. All reported *p* values are 2-sided.

## Results

We studied 49 patients whose characteristics are summarized in Table 1. Twenty-five of the 49 patients (51%) were defined as responders because thermodilution CI increased by > 10% after VE of 500-ml.

**Table 1:** Baseline characteristics of the study population.

Variables	All patients (n = 49)	Responders (n = 25)	Non-responders (n = 24)	*p*-value
Age (years)	67 [60–73]	62 [59–74]	68 [65–71]	0.25
Weight (kg)	79 [67–96]	80 [67–90]	78 [66–100]	0.89
BMI (kg/m^2^)	27.2 [23.6–33.0]	26.5 [22.6–29.2]	27.5 [23.8–32.4]	0.32
Admission SAPS II	62 ± 15	62 ± 17	65 ± 20	0.57
SOFA score	10 [7–14]	10 [7–12]	10 [7–14]	0.54
Male, n (%)	34 (69.4)	19 (76.0)	15 (62.5)	0.30
Mechanical ventilation, n (%)	49 (100)	25 (100)	24 (100)	1.00
**Infection source, n (%)**				
Pneumonia	27 (55)	14 (56)	13 (54)	0.88
Peritonitis	12 (25)	7 (28)	6 (25)	0.93
Meningitis	3 (6)	2 (8)	1 (4)	0.99
Catheter related infections	2 (4)	0 (0)	2 (8)	0.46
Others	5 (10)	2 (8)	3 (12)	0.96
Norepinephrine, n (%)	37 (75.5)	19 (76)	18 (75)	0.93
Norepinephrine (µg kg min^−1^)	0.14 [0.06–0.45]	0.14 [0.05–0.46]	0.13 [0.06–0.47]	0.99

BMI, body mass index; SAPS II, Simplified Acute Physiologic Score; SOFA, Sequential Organ failure Assessment. Data are expressed as mean ± SD, median [25–75 interquartile range], or count.

There were no significant differences in patient characteristics, SOFA score, and norepinephrine between responders and non-responders (Table 1).

### Effect of volume expansion on hemodynamic variables

At baseline, all the tested hemodynamic variables were similar between the two groups (Table 2). VE significantly increased arterial pressures, CVP, and intrathoracic blood volume, and decreased hemoglobin in both groups. CI and stroke volume index increased significantly only in responders after VE, whereas HR and extravascular lung water did not change (Table 2). Arterial and venous pH were not significantly different between responders and non-responders’ groups at baseline, and did not change significantly after VE (Table 2).

**Table 2:** Hemodynamic and acid–base variables before and after 500 ml of volume expansion.

	Before volume expansion	After volume expansion
**Heart rate (beats min**^−**1**^**)**		
Responders	101 ± 26	100 ± 26
Non-responders	99 ± 25	97 ± 26
**Systolic arterial pressure (mmHg)**		
Responders	102 ± 21	119 ± 20^#^
Non-responders	107 ± 29	116 ± 28^#^
**Diastolic arterial pressure (mmHg)**		
Responders	58 ± 12	59 ± 12^#^
Non-responders	55 ± 12	62 ± 10^#^
**Mean arterial pressure (mmHg)**		
Responders	72 ± 13	81 ± 12^#^
Non-responders	72 ± 17	78 ± 17^#^
**Pulse pressure (mmHg)**		
Responders	44 ± 18	57 ± 17^#^
Non-responders	52 ± 21	57 ± 22^#^
**Central venous pressure (mmHg)**		
Responders	13 ± 6	16 ± 6^#^
Non-responders	16 ± 5	19 ± 6^#^
**Intra-thoracic blood volume index (ml m**^−**2**^**)**		
Responders	841 ± 188	951 ± 215^#^
Non-responders	858 ± 236	971 ± 223^#^
**Extravascular lung water index (ml kg**^−**1**^**)**		
Responders	9.1 ± 4.2	9.2 ± 4.3
Non-responders	8.9 ± 3.6	9.1 ± 3.4
**Systemic vascular resistance index (DS m**^−**2**^** cm**^**5**^**)**		
Responders	1702 [1218–2225]	1587 [1220–1990]^#^
Non-responders	1540 [957–1894]	1678 [1123–2096]
**Cardiac index (l min**^−**1**^** m**^−**2**^**)**		
Responders	2.6 [2.1–3.5]	3.46 [2.59–4.16]^#^
Non-responders	2.9 [2.2–3.9]	2.78 [2.19–4.07]
**Stroke volume index (ml m**^−**2**^**)**		
Responders	29.6 ± 10.5	36.6 ± 10.7^#^
Non-responders	33.6 ± 13.6	33.6 ± 12.2
**Hemoglobin (g/dL)**		
Responders	10.3 ± 1.6	9.5 ± 1.3^#^
Non-responders	9.6 ± 1.8	9.1 ± 1.6^#^
**Arterial pH**		
Responders	7.35 [7.28–7.37]	7.34 [7.26–7.36]
Non-responders	7.34 [7.20–7.39]	7.31 [7.21–7.37]
**Central venous pH**		
Responders	7.28 [7.22–7.33]	7.30 [7.22–7.33]
Non-responders	7.30 [7.15–7.34]	7.28 [7.16–7.34]
**Base excess (mmol L**^−**1**^**)**		
Responders	− 6.5 [− 8.9 to − 1.7]	− 6.7 [− 9.5 to − 2.1]^#^
Non-responders	− 4.7 [− 12.4 to − 1.1]	− 5.1 [− 12.1 to − 1.7]^#^

Data are expressed as mean (SD) or median [25–75 interquartile range]. Responders n = 25; Non-responders n = 24.

*p < 0.05 comparisons between responders and non-responders.^#^*p* < 0.05 comparisons between before and after before volume expansion.

### Effect of volume expansion on oxygenation and CO_2_-derived variables

At baseline, there were no significant differences between the responders and non-responders’ groups regarding all the oxygenation and CO_2_-derived variable (Table 3). DO_2_ and ScvO_2_ increased significantly, and OE decreased only in responders’ group after VE. VE significantly reduced ΔPCO_2_ and lactate levels only in the responders’ group (Table 3). VO_2_ and ΔPCO_2_/ΔContO_2_ ratio remained unchanged in both groups after VE.

**Table 3:** Oxygenation and CO_2_-derived variables before and after 500 ml of volume expansion.

	Before volume expansion	After volume expansion
**Central venous oxygen saturation (%)**		
Responders	62 ± 13	68 ± 13^#^*
Non-responders	57 ± 15	56 ± 15
**Oxygen delivery (ml min**^**−1**^** m**^**−2**^**)**		
Responders	382 ± 130	441 ± 134^#^
Non-responders	388 ± 163	368 ± 151
**Arterial oxygen content (mL)**		
Responders	13.4 ± 2.2	12.5 ± 1.8^#^
Non-responders	12.3 ± 2.5	11.8 ± 2.2
**Central venous oxygen content (mL)**		
Responders	8.6 ± 2.2	8.7 ± 2.1*
Non-responders	7.4 ± 2.6	7.1 ± 2.6
**Oxygen consumption (mL min**^**−1**^** m**^**−2**^**)**		
Responders	129.6 ± 44.5	125.8 ± 45.2
Non-responders	144.3 ± 49.5	139.3 ± 51.0
**Oxygen extraction (%)**		
Responders	36.0 ± 12.8	30.4 ± 12.8*^#^
Non-responders	40.0 ± 15.0	41.1 ± 14.9
**PaCO**_**2**_** (mmHg)**		
Responders	38 [33–41]	39 [32–41]
Non-responders	38 [34–40]	38 [33–41]
**PcvCO**_**2**_** (mmHg)**		
Responders	45 [38–49]	44 [36–48]^#^
Non-responders	45 [42–47]	45 [41–50]
**ΔPCO**_**2**_** (mmHg)**		
Responders	7.0 [5.0–9.0]	5.0 [3.0–6.0]^#^
Non-responders	7.0 [5.0–9.0]	6.5 [5.0–9.0]
**ΔPCO**_**2**_**/ΔContO**_**2**_** (mmHg/mL)**		
Responders	1.59 ± 0.53	1.51 ± 0.56
Non-responders	1.60 ± 0.70	1.60 ± 0.49
**Lactate (mmol/L)**		
Responders	2.0 [1.1–3.6]	1.7 [1.0–3.4]^#^
Non-responders	1.5 [1.3–4.7]	1.6 [1.3–4.2]
**Arterial oxygen saturation (%)**		
Responders	96 [92–99]	96 [94–98]
Non-responders	96 [93–97]	96 [94–97]
**PaO**_**2**_**/FiO**_**2**_** ratio (mmHg)**		
Responders	202 [149–294]	211 [157–295]
Non-responders	197 [124–273]	202 [154–265]

PaCO_2_, arterial CO_2_ tension; PcvO_2_, central venous CO_2_ tension; ΔPCO_2_, central venous-to-arterial PCO_2_ difference; ΔContO_2_, arterial-to-venous oxygen content difference; PaO_2_, arterial oxygen tension; FiO_2_, inspiratory oxygen fraction.

Data are expressed as mean (SD) or median [25–75 interquartile range]. Responders n = 25; Non-responders n = 24.

*p < 0.05 comparisons between responders and non-responders.^#^*p* < 0.05 comparisons between before and after before volume expansion.

We observed significant correlations between ΔScvO_2_ and ΔCI (r = 0.42, *p* = 0.003) and between Δ-ΔPCO_2_ and ΔCI (r =  − 0.30, *p* = 0.03) after VE.

In patients with an increase in VO_2_ ≤ 10% (n = 36) after VE, the correlation between ΔScvO_2_ and ΔCI was of 0.62 (*p* < 0.001). Also, in these patients, Δ-ΔPCO_2_ was significantly correlated with ΔCI (r =  − 0.47, *p* = 0.004).

### Ability of ΔScvO_2_ and Δ-ΔPCO_2_ to define fluid responsiveness (increase in CI > 10% after VE)

**Figure 1:**
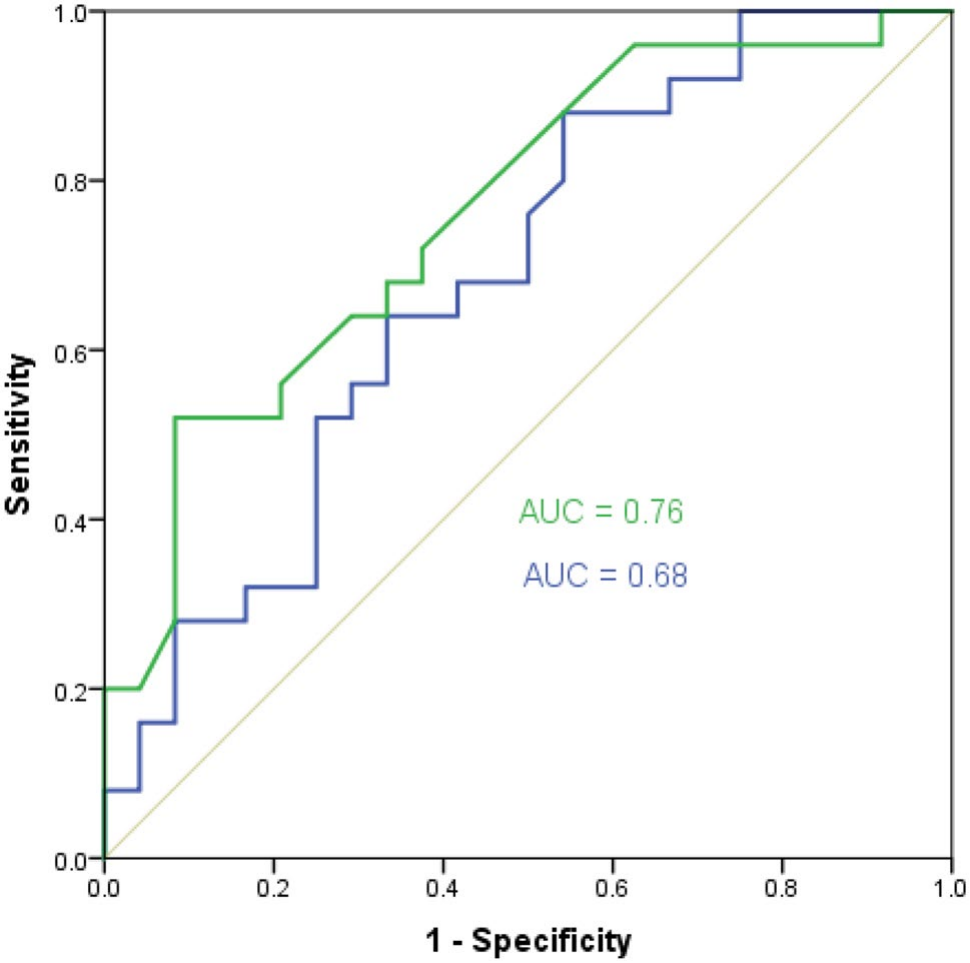
Receiver operating characteristic (ROC) curves showing the ability of the changes in ΔPCO_2_ (Δ-ΔPCO_2_) (green curve), ScvO_2_ (ΔScvO_2_) (blue curve) between before and after 500 mL of volume expansion to define fluid responsiveness (increase in cardiac index > 10% after volume expansion).

The AUC for ΔScvO_2_ was 0.68 (95% CI: 0.53–0.83) (*p* = 0.02) and for Δ-ΔPCO_2_ was 0.76 (95% CI: 0.63–0.89) (*p* < 0.001) (Fig. 1). There were no significant differences between the AUCs for ΔScvO_2_ and Δ-ΔPCO_2_ (*p* = 0.41).

The best cutoff value (according to Youden index) for ΔScvO_2_ was ≥ 3.5% (sensitivity = 64% [95% CI: 42–82%], specificity = 65% [95% CI: 43–84%]), which was lower than its LSC (5%). Taking into account the repeatability (LSC), the best cutoff value was ≥ 7.4% (sensitivity = 56% [95% CI: 35–76%], specificity = 71% [95% CI: 49–87%], PPV = 63% [95% CI: 39–83%], NPV = 61% [95% CI: 41–78%], LR^+^ = 1.8 [95% CI: 0.9–3.7], and LR^−^ = 0.7 [95% CI: 0.4–1.1]).

The best cutoff value (according to Youden index) for Δ-ΔPCO_2_ was ≤ -23.5% (sensitivity = 52% [95% CI: 31–72%], specificity = 87% [95% CI: 68–97%], which was lower than its LSC (36.5%). Taking into account the repeatability (LSC), the best cutoff value was ≤ -37.5% (sensitivity = 32% [95% CI:15–53%], specificity = 92% [95% CI: 73–99%], PPV = 79% [95% CI: 42–97%], NPV = 58% [95% CI: 42–74%], LR^+^ = 3.8 [95% CI: 0.9–16.3], and LR^−^ = 0.7 [95% CI:0.6–1.0]).

**Figure 2:**
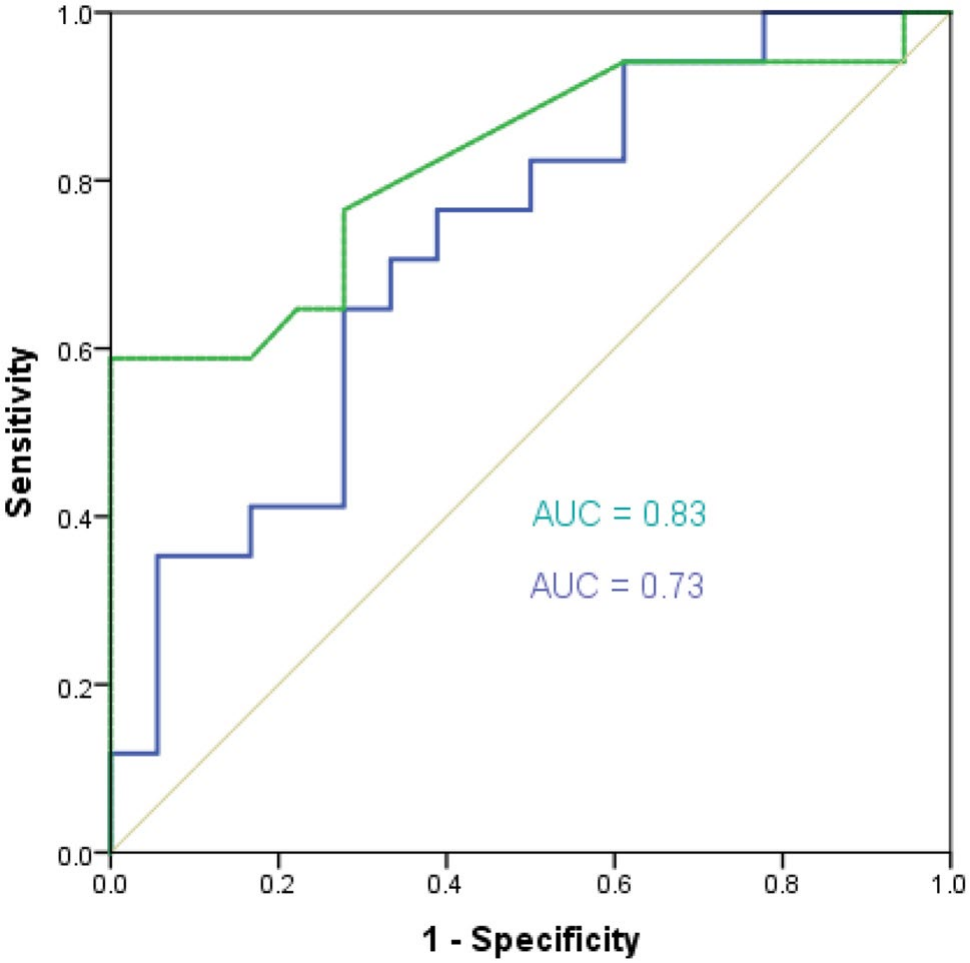
Receiver operating characteristic (ROC) curves showing the ability of the changes in ΔPCO_2_ (Δ-ΔPCO_2_) (green curve), ScvO_2_ (ΔScvO_2_) (blue curve) between before and after 500 mL of volume expansion to define fluid responsiveness (increase in cardiac index > 10% after volume expansion) in the subgroup of patients with an increase in oxygen consumption ≤ 10%.

In patients with an increase in VO_2_ ≤ 10%, the AUC for ΔScvO_2_ was 0.73 (95% CI: 0.57–0.90) (*p* = 0.006) and for Δ-ΔPCO_2_ was 0.83 (95% CI: 0.69–0.97) (*p* < 0.001) (Fig. 2). There was no significant difference between the AUCs for ΔScvO_2_ and Δ-ΔPCO_2_ (*p* = 0.41).

The best cutoff value (according to Youden index) for ΔScvO_2_ was ≥ 8.1% (sensitivity = 65% [95% CI: 38–86%], specificity = 74% [95% CI: 49–91%]), PPV = 73% [95% CI: 47–91%], NPV = 65% [95% CI: 40–85%], LR^+^ = 2.5 [95% CI: 1.1–5.6], and LR^−^ = 0.5 [95% CI: 0.2–1.0]).

The best cutoff value (according to Youden index) for Δ-ΔPCO_2_ was ≤ -25% (sensitivity = 59% [95% CI: 33–82%], specificity = 89% [95% CI: 65–99%], which was lower than its LSC (36.5%). Taking into account the repeatability (LSC), the best cutoff value was ≤  − 37.5% (sensitivity = 41% [95% CI: 18–67%], specificity = 100% [95% CI: 59–100%], PPV = 100% [95% CI: 62–100%], NPV = 60% [95% CI: 40–78%], LR^+^ = ∞, and LR^−^ = 0.60 [95% CI: 0.4–0.9]).

### Characteristics of patients with tissue hypoxia (ΔVO_2_ > 10% after VE)

At baseline, ΔPCO_2_/ΔContO_2_ ratio was significantly higher in patients with ΔVO_2_ > 10% (tissue hypoxia) induced by volume expansion compared to patients with ΔVO_2_ ≤ 10% (2.03 [1.73–2.27] vs. 1.39 [1.00–1.71] mmHg/mL, *p* = 0.03, respectively). We did not observe significant differences between patients with ΔVO_2_ > 10% and ΔVO_2_ ≤ 10% regarding baseline lactate levels (2.35 [1.02–3.97] vs. 1.70 [1.40–3.10] mmol/L, *p* = 0.89, respectively) and baseline ScvO_2_ levels (60 ± 17% vs. 59 ± 13%, *p* = 0.78, respectively).

**Figure 3:**
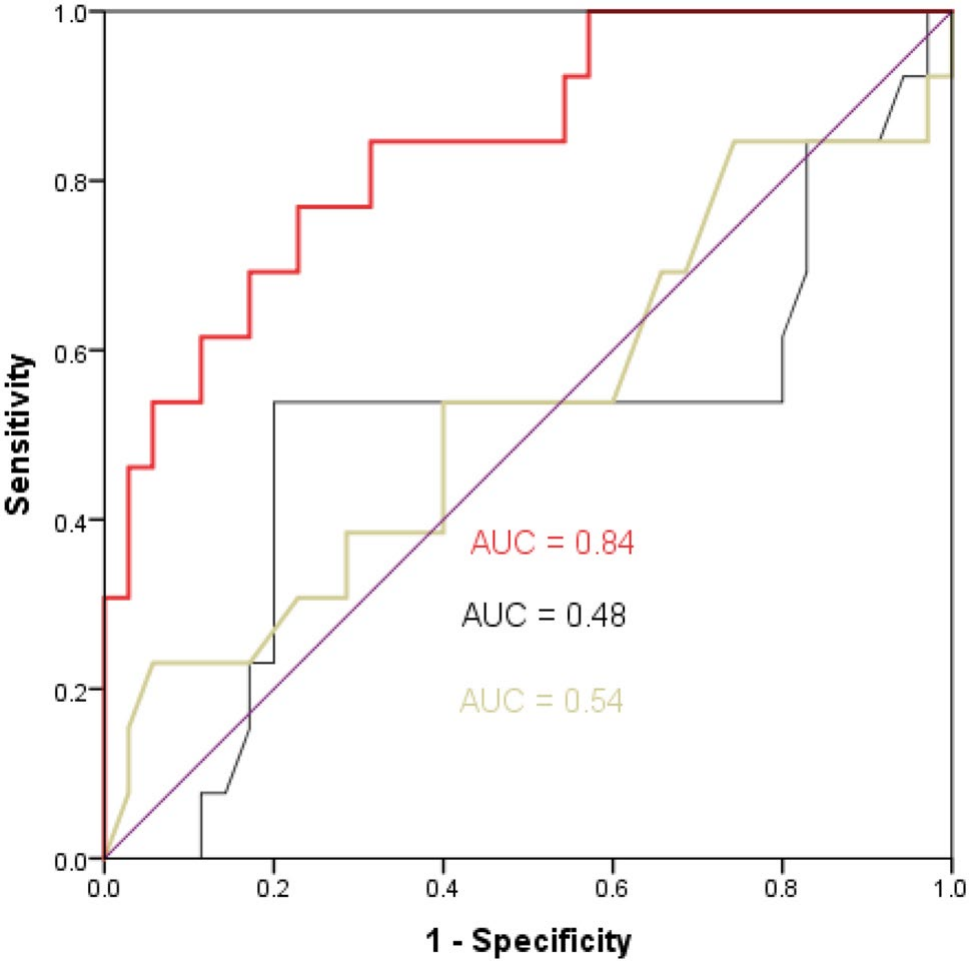
Receiver operating characteristic (ROC) curves showing the ability of baseline ΔPCO_2_/ΔContO_2_ (red curve), ScvO_2_ (yellow curve), and lactate (black curve) to predict an increase in oxygen consumption > 10% after 500 mL of volume expansion.

The AUCs of baseline lactate and ScvO_2_ values to predict ΔVO_2_ > 10% were 0.48 (95% CI: 0.28–0.69) (*p* = 0.89) and 0.54 (95% CI: 0.33–0.74) (*p* = 0.72), respectively. The AUC of baseline ΔPCO_2_/ΔContO_2_ ratio to predict ΔVO_2_ > 10% after volume expansion was 0.84 (95% CI: 0.71–0.96) (*p* < 0.001) (Fig. 3). The best cutoff value (according to Youden index) for baseline ΔPCO_2_/ΔContO_2_ ratio was > 1.70 (sensitivity = 77% [95% CI: 46–95%], specificity = 77% [95% CI: 60–90%]), PPV = 90% [95% CI: 73–98%], NPV = 55% [95% CI :31–78%], LR^+^ = 3.4 [95% CI: 1.7–6.6], and LR^−^ = 0.3 [95% CI: 0.1–0.8]).

## Discussion

The main findings of our study are: (1) in the whole population Δ-ΔPCO_2_ induced by VE has an acceptable ability to define fluid responsiveness, but not ΔScvO_2_; (2) The abilities of Δ-ΔPCO_2_ and ΔScvO_2_ to define fluid responsiveness improved when we considered only patients in whom changes in VO_2_ were minimal (ΔVO_2_ ≤ 10%) after VE, i.e., patients without tissue hypoxia; 3) Baseline ΔPCO_2_/ΔContO_2_ ratio has a good ability to predict the presence of tissue hypoxia (increases in VO_2_ > 10% after VE).

Applying the modified Fick method to CO_2_, venous-to-arterial PCO_2_ difference reflects cardiac output. Several experimental studies have demonstrated the primary role of decreased tissue blood flow in the increased venous-to-arterial PCO_2_ gap^8,21,22^. Similarly, a mathematical model analysis has confirmed that blood flow represents the major determinant in the elevation of venous-to-arterial PCO_2_ gap^23^. A rise in mixed venous-to-arterial PCO_2_ gap that was directly linked to a decrease in cardiac output has been observed in different types of circulatory failure including septic shock^24,25^. Mecher et al. found a significant negative correlation between the changes in cardiac output and mixed venous-to-arterial PCO_2_ gap after fluid resuscitation in septic shock patients (r =  − 0.42, *p* < 0.01)^24^. In post-cardiac surgery sedated and mechanically ventilated patients with cardiac index < 2.5 L/min/m^2^, ΔCI induced by VE (500-mL bolus of crystalloid given over 30 min) was found to be significantly correlated with Δ-ΔPCO_2_ (r =  − 0.53, *p* = 0.001)^13^. However, the AUC for Δ-ΔPCO_2_ to define fluid responsiveness was not determined in that study.

The correlation between Δ-ΔPCO_2_ and ΔCI induced by VE was weaker in our septic patients than what was observed in post-cardiac surgery patients^13^. Also, the ability of Δ-ΔPCO_2_ to define fluid responsiveness was not robust (AUC = 0.76). These findings could be explained by several factors. First, the relationship between ΔPCO_2_ and CI is curvilinear^9^, which means that the magnitude of changes in ΔPCO_2_ is more pronounced at low CI than at normal or high CI. Second, the relationship between CO_2_ content and PCO_2_, which is curvilinear rather than linear, is influenced by many factors such as the degree of metabolic acidosis, hematocrit, and oxygen saturation (Haldane effect)^9,26^. However, we believe that this factor is unlikely to have occurred in our patients. Although base excess and hemoglobin significantly decreased in both groups (responders and non-responders) and ScvO_2_ increased only in the responders’ group (Tables 2, 3) after VE, it is unlikely that these clinically irrelevant changes could have affected the PCO_2_/CO_2_ content relationship. If these changes had affected the PCO_2_/CO_2_ content relationship, it would have resulted in an increase in ΔPCO_2_ in both groups. Third, in situations of tissue hypoxia with VO_2_/DO_2_ dependency phenomenon and anaerobic CO_2_ production, the rise in CI would increase VO_2_ and VCO_2_. This would attenuate the decrease in ΔPCO_2_ related to the increase in blood flow^9,27^. We believe that this factor may have contributed to the reduction in the performance of Δ-ΔPCO_2_ (AUC = 0.76) in defining an increase in CI > 10% induced by VE (fluid responsiveness) in the overall population. When we excluded patients with tissue hypoxia, patients with an increase in VO_2_ ≤ 10% (VO_2_/DO_2_ independency), we observed an improvement in the ability of Δ-ΔPCO_2_ to define fluid responsiveness with a very good AUC of 0.83 (Fig. 2). A decrease in Δ-ΔPCO_2_ of more or equal than 37.5% induced by VE allowed discrimination between responders and non-responders with a PPV of 100%. Also, the correlation between Δ-ΔPCO_2_ and ΔCI was higher than in the overall population.

Venous oxygen saturation is a global marker of adequacy between oxygen consumption and oxygen supply^28^. Therefore, changes in venous oxygen saturation reflect changes in the balance between VO_2_ and DO_2_ and indicate tissue oxygenation. Giraud et al. observed, in 30 cardiogenic shock or postoperative cardiac surgery patients, that ΔScvO_2_ was significantly correlated with ΔCI induced by a bolus of 500 mL of normal saline administered over 10-min (r = 0.67, *p* < 0.001)^14^. Also, ΔScvO_2_ had an excellent ability to define an increase in CI ≥ 15% after VE (fluid responsiveness) with an AUC of 0.90. Our findings are different from those reported by Giraud et al.^14^. We observed in our whole septic population a weaker correlation between ΔScvO_2_ and ΔCI (r = 0.42), and ΔScvO_2_ had a poor ability to discriminate between responders and non-responders. In the subgroup of patients without tissue hypoxia (no significant increase in VO_2_, or VO_2_/DO_2_ independency), even though the correlation between ΔScvO_2_ and ΔCI improved, the ability of ΔScvO_2_ to characterize fluid responsiveness was not good. The main explanation of the discrepancies between our findings and those of Giraud et al.^14^ is that the patient populations are different. As has been previously described, venous oxygen saturation may not be a good indicator of tissue oxygenation in the setting of sepsis, due to microcirculatory shunting and mitochondrial dysfunction that can result in oxygen extraction abnormalities^29^.

It has been suggested that ΔPCO_2_/ΔContO_2_ ratio, considered as a surrogate of the respiratory quotient, can be used as a marker of global tissue hypoxia in critically ill patients^15,16,30^. In our study, we found that baseline ΔPCO_2_/ΔContO_2_ value was significantly higher in patients with global tissue hypoxia (defined as an increase in VO_2_ > 10% after VE) than those without global tissue hypoxia. Also, baseline ΔPCO_2_/ΔContO_2_ ratio had a very good ability to predict the presence of VO_2_/DO_2_ dependency (global tissue hypoxia). Our results confirmed our previous findings^15^ and those reported by Monnet et al.^16^, who observed excellent predictability of baseline ΔPCO_2_/ΔContO_2_ value for tissue hypoxia (AUCs = 0.96 and 0.94, respectively). Baseline lactate and ScvO_2_ levels had poor ability to predict VO_2_/DO_2_ dependency (global tissue hypoxia) in our study. This finding is in line of what we observed previously in septic shock patients^15^.

To the best of our knowledge, our study is the first to investigate the role of Δ-ΔPCO_2_ and ΔScvO_2_ in defining fluid responsiveness in septic patients. Our findings are valuable as they can be integrated in a clinical algorithm and used by the bedside provider as part of the assessment of fluid responsiveness. These values are readily available as patients in septic shock usually have arterial and central venous catheters inserted. After measuring O_2_ content and CO_2_ partial pressures, the ΔPCO_2_/ΔContO_2_ ratio can help the provider recognize patients without tissue hypoxia. In these patients, ΔPCO_2_ can be measured before and after VE and be used to appreciate if the latter has resulted in a significant increase in CI and to guide further fluid resuscitation when cardiac output monitoring is not available.

Our study presents several limitations. First, it is a single-center study, so the results might not universally apply. Second, we used central venous samples instead of mixed venous to assess oxygen and CO_2_-derived variables, limiting its accuracy. However, we were interested in the changes in these variables induced by fluid challenge rather than their absolute values. Moreover, it has been shown that calculating the oxygen and CO_2_-derived variables from the central venous blood permitted the detection of global tissue hypoxia in critically ill patients^15,16^. Third, our patients were sedated and mechanically ventilated with stable oxygen consumption; thus, our findings might not apply to spontaneously breathing patients with varying oxygen demands. Finally, our study was not sufficiently powered for subgroup analyses; thus, our findings need to be replicated in a future study with larger sample size.

## Conclusions

In sedated and mechanically ventilated septic patients with no signs of tissue hypoxia, Δ-ΔPCO_2_ is a reliable parameter to define fluid responsiveness and can be used in the absence of CI measurement. Baseline ΔPCO_2_/ΔContO_2_ ratio could help the physician recognize the presence of tissue hypoxia.

## Abbreviations

CO_2_: Carbon dioxide
VO_2_: Oxygen consumption
DO_2_: Oxygen delivery
∆PCO_2_: Venous-to-arterial carbon dioxide tension difference
ΔContO_2_: Arterial-to-venous oxygen content difference
PaCO_2_: Partial arterial carbon dioxide tension
PcvCO_2_: Central venous carbon dioxide tension
ScvO_2_: Central venous oxygen saturation
SaO_2_: Arterial oxygen saturation
PaO_2_: Partial arterial oxygen tension
PvO_2_: Partial venous oxygen tension
Hb: Hemoglobin
CI: Cardiac index
LSC: Least significant change
AUC: Area under the curve
ROC: Receiver operating characteristic
PPV: Positive predictive value
NPV: Negative predictive value
LR: Likelihood ratio
VE: Volume expansion
HR: Heart rate
ICU: Intensive care unit

## References

[1] Michard F, Teboul JL (2002). Predicting fluid responsiveness in ICU patients: a critical analysis of the evidence. Chest.

[2] Vincent JL, Weil MH (2006). Fluid challenge revisited. Crit. Care Med..

[3] Cecconi M, Parsons AK, Rhodes A (2011). What is a fluid challenge?. Curr. Opin. Crit. Care.

[4] Monnet X, Rienzo M, Osman D (2006). Passive leg raising predicts fluid responsiveness in the critically ill. Crit. Care Med..

[5] Monnet X, Osman D, Ridel C, Lamia B, Richard C, Teboul JL (2009). Predicting volume responsiveness by using the end-expiratory occlusion in mechanically ventilated intensive care unit patients. Crit. Care Med..

[6] Mallat J, Meddour M, Durville E (2015). Decrease in pulse pressure and stroke volume variations after mini-fluid challenge accurately predicts fluid responsiveness. Br. J. Anaesth..

[7] Mahjoub Y, Lejeune V, Muller L (2014). Evaluation of pulse pressure variation validity criteria in critically ill patients: a prospective observational multicentre point-prevalence study. Br. J. Anaesth..

[8] Vallet B, Teboul JL, Cain S, Curtis S (2000). Venoarterial CO(2) difference during regional ischemic or hypoxic hypoxia. J. Appl. Physiol..

[9] Mallat J, Lemyze M, Tronchon L, Vallet B, Thevenin D (2016). Use of venous-to-arterial carbon dioxide tension difference to guide resuscitation therapy in septic shock. World J. Crit. Care Med..

[10] Mallat J, Pepy F, Lemyze M (2014). Central venous-to-arterial carbon dioxide partial pressure difference in early resuscitation from septic shock: a prospective observational study. Eur. J. Anaesthesiol..

[11] Vallée F, Vallet B, Mathe O (2008). Central venous-to-arterial carbon dioxide difference: an additional target for goal-directed therapy in septic shock?. Intensive Care Med..

[12] Cuschieri J, Rivers EP, Donnino MW (2005). Central venous-arterial carbon dioxide difference as an indicator of cardiac index. Intensive Care Med..

[13] Yazigi A, Abou-Zeid H, Haddad F, Madi-Jebara S, Hayeck G, Jabbour K (2010). Correlation between central venous-arterial carbon dioxide tension gradient and cardiac index changes following fluid therapy. Ann. Card Anaesth..

[14] Giraud R, Siegenthaler N, Gayet-Ageron A, Combescure C, Romand JA, Bendjelid K (2011). ScvO(2) as a marker to define fluid responsiveness. J Trauma.

[15] Mallat J, Lemyze M, Meddour M (2016). Ratios of central venous-to-arterial carbon dioxide content or tension to arteriovenous oxygen content are better markers of global anaerobic metabolism than lactate in septic shock patients. Ann. Intensive Care..

[16] Monnet X, Julien F, Ait-Hamou N (2013). Lactate and venoarterial carbon dioxide difference/arterial–venous oxy-gen difference ratio, but not central venous oxygen saturation, predict increase in oxygen consumption in fluid responders. Crit. Care Med..

[17] Dellinger RP, Levy MM, Rhodes A (2013). Surviving sepsis campaign: international guidelines for management of severe sepsis and septic shock: 2012. Crit. Care Med..

[18] DeLong ER, DeLong DM, Clarke-Pearson DL (1988). Comparing the areas under two or more correlated receiver operating characteristic curves: a nonparametric approach. Biometrics.

[19] Mallat J, Lazkani A, Lemyze M (2015). Repeatability of blood gas parameters, PCO2 gap, and PCO2 gap to arterial-to-venous oxygen content difference in critically ill adult patients. Medicine (Baltimore).

[20] Ray P, Le Manach Y, Riou B, Houle TT (2010). Statistical evaluation of a biomarker. Anesthesiology.

[21] Nevière R, Chagnon JL, Teboul JL, Vallet B, Wattel F (2002). Small intestine intramucosal PCO(2) and microvascular blood flow during hypoxic and ischemic hypoxia. Crit. Care Med..

[22] Dubin A, Murias G, Estenssoro E (2002). Intramucosal-arterial PCO2 gap fails to reflect intestinal dysoxia in hypoxic hypoxia. Crit. Care.

[23] Gutierrez G (2004). A mathematical model of tissue-blood carbon dioxide exchange during hypoxia. Am. J. Respir. Crit. Care Med..

[24] Mecher CE, Rackow EC, Astiz ME, Weil MH (1990). Venous hypercarbia associated with severe sepsis and systemic hypoperfusion. Crit. Care Med..

[25] Bakker J, Vincent JL, Gris P, Leon M, Coffernils M, Kahn RJ (1992). Veno-arterial carbon dioxide gradient in human septic shock. Chest.

[26] Teboul JL, Scheeren T (2017). Understanding the Haldane effect. Intensive Care Med..

[27] Lamia B, Monnet X, Teboul JL (2006). Meaning of arterio-venous PCO2 difference in circulatory shock. Minerva Anestesiol..

[28] Squara P (2014). Central venous oxygenation: when physiology explains apparent discrepancies. Crit. Care.

[29] Vallet B, Pinsky MR, Cecconi M (2013). Resuscitation of patients with septic shock: please "mind the gap"!. Intensive Care Med..

[30] Mekontso-Dessap A, Castelain V, Anguel N (2002). Combination of venoarterial PCO2 difference with arteriovenous O2 content difference to detect anaerobic metabolism in patients. Intensive Care Med..

